# Supplementary material for: Optimization of the Design Configuration and Operation Strategy of Single-Pass Seawater Reverse Osmosis
Source: Membranes (Basel). 2022 Nov 15;12(11):1145. doi: 10.3390/membranes12111145 (PMC9692277; doi:10.3390/membranes12111145)
Supplement: Supplementary file 1 [file membranes-12-01145-s001.zip › membranes-2018281-supplementary.pdf]

Supplementary materials

Optimization of the Design Configuration and Operation Strategy of Single-Pass Seawater Reverse Osmosis

Seung Ji Lim [1,2], Seo Jin Ki [3,*] , Jae-Lim Lim [4], Kyunghyuk Lee [4], Jihye Kim [4], Jeongwoo Moon [1] and Joon Ha Kim [1]

[1]*School of Earth Sciences and Environmental Engineering, Gwangju Institute of Science and Technology (GIST), Gwangju 61005, Republic of Korea*

[2]*Center for Water Cycle Research, Korea Institute of Science and Technology (KIST), Seoul 02792, Republic of Korea*

[3]*Department of Environmental Engineering, Gyeongnam National University of Science and Technology, 33 Dongjin-ro, Jinju 52725, Republic of Korea*

[4]*Korea Water Resources Corporation (K-Water), Daejeon, Republic of Korea*

[*]*Correspondence: seojinki@gnu.ac.kr; Tel.: +82-55-751-3341*

Table S1. Parameter values for the process constant permeate mode.

Parameter	Value
SWRO model operating conditions	
Target recovery ratio (%)	38–42
Operation duration (days)	365
Membrane element properties	
Intrinsic membrane resistance R_m (m^{-1})	2.89×10^{14}
Salt permeability coefficient B (m/s)	9.36×10^{-9}
Spacer thickness H (m)	8.64×10^{-4}
Membrane channel width w (m)	37
Membrane channel length L (m)	1
Number of membrane elements in a pressure vessel	7
Hydrodynamic properties	
Fouling potential of feedwater, k_{fp} (m^{-2})	5.5×10^{11}
Hydraulic dispersion coefficient D (−)	9.6×10^{-9}
Friction coefficient due to the membrane spacer k_{fr} (−)	8

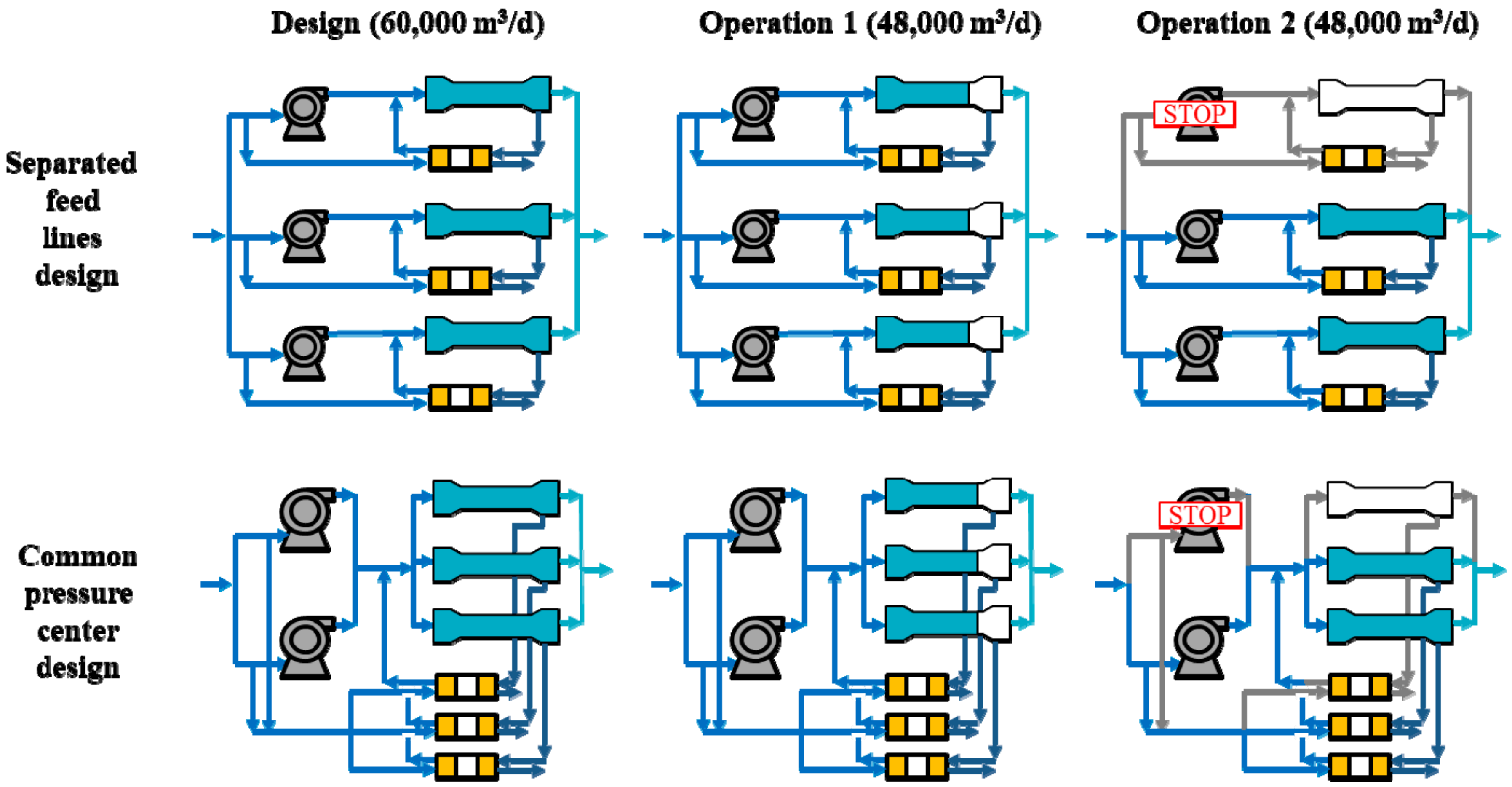

Figure S1. Graphical summary of design and operation configuration employed in this study.
